# Re-thinking the preclinical development of GBM therapeutics

**DOI:** 10.18632/oncoscience.396

**Published:** 2018-02-23

**Authors:** Érika Cosset, Sara M. Weis, David A. Cheresh

**Affiliations:** Department of Internal Medicine Specialities, Centre of Translational Onco-Hematology, Medical School, University of Geneva, Geneva, Switzerland

**Keywords:** Glut3, cancer stem cells, glioblastoma, glucose metabolism, integrin

Glioblastoma multiforme (GBM) remains one of the most incurable cancers. Although the genomic era has produced massive quantities of data in an attempt to characterize its molecular drivers[1, 2], these advances have yet to be effectively translated into clinical impact. In fact, several studies have identified the presence of all three GBM molecular subtypes within a single patient tumor[3, 4], illustrating the complexity of designing personalized medicine approaches. In addition to the strong intra/inter-tumoral heterogeneity, the inability of targeted therapies to achieve long-term remissions is likely a function of multiple complicating factors, including the presence of glioblastoma stem cells, redundant signaling pathways, the unique infiltrative nature of GBM cells, and difficulties associated with drug delivery across the blood-brain-barrier. New strategies to match patients to molecularly targeted therapies will also need to overcome these challenges imposed by the challenging GBM microenvironment within the brain.

The clinical development and testing of the αv integrin antagonist cilengitide provides an interesting case study to show how a personalized medicine approach may be able to resurrect a therapeutic that failed to provide a survival benefit in an “unselected” GBM patient population. Cilengitide (EMD 121974), is a cyclic peptide designed to block the function of αvβ3 and αvβ5 integrins that had been implicated as key drivers of tumor angiogenesis and glioblastoma progression[5]. While higher levels of αvβ3 were associated with a modest survival benefit for cilengitide, αvβ3 expression status alone did not correlate with patient outcome[6]. Although a number of patients showed an unexplained high sensitivity to the drug and achieved long-term stable disease, cilengitide failed to meet its overall survival endpoints in phase II-III clinical trials and its clinical development was halted[7]. Considering the wealth of studies pointing to integrin αvβ3 as a key driver of GBM progression, we and others involved in the preclinical testing of integrin antagonists were disappointed and perplexed when cilengitide did not provide a more significant benefit in man.

We therefore reconsidered whether all GBM tumors are equally addicted to integrin αvβ3 during progression. Remarkably, we discovered that sensitivity to αvβ3 blockade could not simply be predicted by high αvβ3 expression, but rather on a state of addiction to the high-affinity glucose transporter, Glut3, that provides tumors with a critical glucose-uptake advantage in the nutrient-poor environment of the brain[8]. Mechanistically, we identified integrin αvβ3 signaling through PAK4 and YAP/TAZ as being necessary and sufficient to promote the expression of Glut3. Accordingly, individual patient-derived GBM tumors that showed sensitivity to Glut3 knockdown showed sensitivity to not only cilengitide and LM609 (a monoclonal antibody antagonist of αvβ3), but also to drugs that inhibit PAK4 and YAP/TAZ.

Furthermore, we identified a genetic signature capable of predicting which GBM tumors would show this Glut3-addicted phenotype. In particular, we found that GBM tumors with markers defining the proneural and classical molecular subtypes were addicted to αvβ3/Glut3. In contrast, tumors with markers defining the mesenchymal subtype were not addicted, even when they displayed high expression of both αvβ3 and Glut3. This distinction highlights how functionally relevant markers can be incorporated into molecular classification approaches to predict which subsets of patients may respond to a given drug, providing important inclusion criteria for clinical trials. Such an analysis to account for durable responses in individual patients will provide a framework for setting up new molecularly defined clinical trials.

Our study also highlights the value of patient-derived gliomaspheres to reflect GBM heterogeneity and addiction status. Indeed, we found the established GBM cell lines U87MG, LN229, and LN18 to be highly sensitive to agents targeting αvβ3/PAK4-YAP/TAZ signaling axis, whereas only a subpopulation of patient-derived GBM stem cell models showed sensitivity. Although the nearly universal effect of cilengitide on established GBM cell lines in vitro and in vivo generated great enthusiasm for its clinical development, only some patients responded to this therapy. In fact, we observed a paradoxical enhancement of viability for several of the “non-addicted” GBM stem cells treated with αvβ3 antagonists. The highly variable response to cilengitide among our panel of patient-derived GBM stem cell models may account for why this drug failed to prolong overall survival in a non-selected GBM patient population. Although our sample size is relatively limited, we predict that less than a quarter of GBM tumors would show sensitivity to cilengitide and that progression may even be accelerated in certain patients. Going forward, the challenge will be to develop rapid and feasible genetic or molecular screens to identify αvβ3/Glut3 addiction in living patients, and to optimize a CLIA-certified screening process to reliably predict responders.

Taken together, our recent study highlights new opportunities to exploit the analysis of “big data” to achieve advances in precision medicine and reinforces the importance of profiling individual tumors. Using cilengitide as an example, we suggest that other “failed” drugs may be reconsidered using patient-derived models, allowing them to advance to a more defined style of GBM clinical trial that relies on molecularly-defined inclusion/exclusion criteria to identify a potentially small population of responders.
